# Heard, known, and safe in general practice? An interview study with patients with severe and persistent mental illness

**DOI:** 10.3399/BJGPO.2021.0201

**Published:** 2022-03-23

**Authors:** Nancy Jennifer Sturman, Ryan Williams, Marianne Wyder, Johanna Lynch

**Affiliations:** General Practice Clinical Unit 1, Royal Brisbane Hospital, Brisbane, QLD, Australia; 2 Stonewall Medical Centre, Windsor, QLD, Australia; 3 Metro South Addiction and Mental Health Services, Brisbane, QLD, Australia

**Keywords:** Australia, general practice, mental disorders, mental health, motivation, physician–patient relations, referral and consultation, primary healthcare

## Abstract

**Background:**

Although GPs provide care to many patients with severe and persistent mental illness, the role and skillset of the GP in this space are contested. Patients are less satisfied with GP care of mental health than physical health issues.

**Aim:**

To explore patient expectations and experiences of GP roles in their mental health, and identify opportunities for improving mental health care in general practice.

**Design & setting:**

Patient participants were recruited from community mental health clinics in Brisbane, Australia.

**Method:**

Individual semi-structured interviews were conducted with a convenience sample of patients. Interviews were audio-recorded and transcribed professionally. The authors conducted an inductive thematic analysis, attending to participant vulnerability and reflexivity.

**Results:**

Sixteen interviews were conducted by one author (RW), with an average duration of 29 minutes. Three overarching themes were identified: being heard, being known, and being safe. Participants greatly valued ‘good GPs’ who were able to detect early signs of relapse, and with whom they came to feel heard, known, and safe over time. Experiences of perfunctory, hurried care and avoidance of mental health issues were also reported. Many participants were uncertain whether GP training in mental health was sufficient to keep them safe. Patients may suspect GPs who predominantly engage with their physical health to have negative attitudes to mental illness.

**Conclusion:**

Some GPs play central roles in patients’ mental health care. Barriers for others need further exploration, and may include time, confidence, and/or expertise. Findings challenge GPs to engage more actively and effectively with these patients in their general practice consultations.

## How this fits in

Many GPs care for people who suffer from the impacts of severe and persistent mental illness, and indeed this has been described as core business for general practice. This study's findings help to clarify the GP’s role with these patients by showing that GPs with ongoing relationships can provide experiences of being heard, known, and safe, and detect early signs of relapse of mental illness. On the other hand, patients may also experience perfunctory, transactional, and/or hurried care, and avoidance of mental health issues, leading them to question the competence, attitudes, and interest of GPs regarding their symptoms, concerns, and goals. Findings challenge those working in general practice to realise the opportunities afforded by regularly scheduled consultations to support patients in realistically hopeful journeys of healing and personal connection.

## Introduction

Despite the high prevalence of mental health issues in general practice consultations^1^ and reports that many mental health consumers and carers use their GP as their primary mental health support,^2^ the role of the GP in the care of patients with severe and persistent mental illness remains contested.

On the one hand, it has been argued that the expertise and skillset of GPs is inadequate to provide adequate care for these clients or patients (henceforth referred to simply as 'patients') without additional psychiatric training.^3,4^ On the other hand, the increasing recognition of the role of complex trauma, including traumatic health care, has highlighted the value of generalist, relationship-based approaches to the whole person.^5,6^ Such approaches, which are oriented towards realistically hopeful journeys of recovery,^7,8^ can usefully complement the disease- and medication-focused approaches of much current psychiatry. Care of people with severe mental illness has indeed been described as core business for general practice,^9^ and GPs are well placed to integrate and coordinate the multiple community services with which many patients are engaged, a well-known difficulty in delivering health and social care to vulnerable clients with complex needs.^10–12^

Patients who are not sick enough to require hospital inpatient care, but not well enough to manage without substantial monitoring and support in the community (recently dubbed 'the missing middle')^2,10^ often present in general practice. A recent Australian survey study, conducted by the investigators, of patients attending community mental health services found that 85% of responders had a regular GP, and 33% had consulted a GP at least 10 times in the preceding 12 months.^13^ The literature suggests that patients vary in their levels of satisfaction with general practice care.^14–16^ In a recent survey study by the authors of the present article, 88% of responders reported being satisfied with their GP’s care, and satisfaction was higher in patients who had a regular GP and/or regular general practice.^13^ Fewer responders were satisfied with their GP’s focus on their mental health (76%) compared with their physical health (95%).^13^ It is important, therefore, to understand patient expectations and experiences of GP roles in their mental health, and to identify opportunities for improving care.

## Method

Patients who had completed a survey about their access to general practice care were invited by RW to participate in an individual, semi-structured, face-to-face interview, if they had provided consent on the survey. The hard copy surveys (see Supplementary File 1 for details) were completed between August and October 2017 while participants were waiting for community mental health clinic appointments in Brisbane, Australia. A flyer about the surveys was displayed at reception, and assistance to complete survey items was provided by RW as requested. RW introduced himself as a GP with an interest in patient experiences of general practice care. Surveys were completed by 82 participants, and these results are reported elsewhere.^13^

The interviews were conducted in a private room in the clinic by RW either immediately after patient appointments or at a subsequent appointment. Although RW attempted to actively follow up all survey responders who consented to an interview (unless they appeared agitated or otherwise acutely unwell), a small number were lost to follow-up. Responders were more likely to be lost to follow-up if they elected to defer their interview to a subsequent appointment. No record was kept of patients who declined to participate or were lost to follow-up. An AUD $25 gift voucher was provided to interview participants. The interview guide focused on participant experiences and expectations of GP care, including any concerns indicated in their survey responses (see Supplementary File 2 for interview guide). Interviews were audio-recorded and transcribed. The first three transcripts were reviewed by RW and NS before completion of all interviews, and minor modifications were made to the interview process.

Two authors (RW and NS) read all transcripts independently, and NS completed first cycle descriptive coding of the full dataset, using participants’ own words (in vivo coding) for many of the initial codes.^17^ Higher order codes and categories^17^ were developed and refined by the authors, using a process of constant comparison with the data, over the course of a total of eight face-to-face meetings, each with two or more of the authors, between 2019 and 2020. No a priori codes or categories were used. All meetings included NS, and followed the completion of data collection. The categories were structured into themes over the course of the analysis. ATLAS.ti 9 software was used to manage the data.

The theoretical approach was that of constructivist realism,^18^ which accepts that meaning is socially constructed, but also that participant accounts are linked with social, external, and personal worlds which have underlying patterns and uniformities. The authors were aware of the power differential between participants and the interviewer, even though no authors had ever been involved in participant care. Three authors (NS, JL, RW) were GPs with a specific clinical interest in patients with severe and persistent mental illness. These authors paid close attention to their own responses to the data, and discussed these over several meetings, in order to set them aside and attend more fully to the participants' 'voices'.^19^ MW is a social worker employed as a research fellow in a public mental health service. Three authors (NS, MW, JL) are female, and had previous experience in qualitative research. RW was supervised by NS.

## Results

Sixteen interviews were conducted in 2017 (average duration 29 minutes, range 21–40 minutes). Data saturation appeared to be achieved after 12 interviews. Table 1 shows participant demographic information. A carer or partner was also present at two interviews, but neither joined the conversation.

**Table 1.** Participant demographic information (*n* = 16)

Characteristic			
**Sex**, *n* (%)	Female = 9 (56)	Male = 7 (44)	Other = 0
**Age**, years	Mean = 39	Range = 25 to 51	
**Country of birth,** ***n*** **(%)**	Australia = 12 (75)	Other^a^ = 4 (25)	
**Main source of income**, *n* (%)	Government pension =13 (81)	Full-time employment =3 (19)	
**Accommodation,** ***n*** **(%)**	Stable = 14 (88)	Lives alone = 6 (38)	
	Unstable = 2 (13)	Lives with others = 10 (63)	
**Education completed**, *n* (%)	Secondary school = 9 (56)	University = 7 (44)	
**Mental health diagnoses** (self-reported),^b^ *n* (%)	Anxiety = 10 (63)	Nil = 1 (6)^c^	
Psychotic illness = 7 (44)	Depression = 9 (56)	PTSD = 3 (19)	
Bipolar affective disorder = 4 (25)	Borderline personality disorder = 5 (31)		
**Currently subject to compulsory psychiatric treatment** (self-reported), *n* (%)	No = 13 (81)	Yes = 3 (19)	
**Regular GP**, *n* (%)	Yes = 14 (87)	No = 2 (14)	
**Number of consultations with any GP in past 12 months**, *n* (%)	Nil = 01 to 3 = 4 (25)	4 to 6 = 3 (19)7 to 9 = 2 (13)≥10 = 7 (44)	

^a^Other = New Zealand, Republic of South Africa, Papua New Guinea, Pacific Islands. ^b^Multiple diagnoses (2–6 diagnoses each) were reported by 12 participants. ^c^This patient indicated that his only mental health issue was 'loneliness'. He was subject to compulsory psychiatric treatment.

PTSD = post-traumatic stress disorder.

Patient expectations and experiences of GP roles in their mental health are presented below using the three overarching themes: being heard, being known, and being safe. Pseudonyms are used for all participants.

### Being heard

Participants emphasised the importance of being heard, and were critical of GPs who appeared not to listen:

*'This GP just sort of acted like he knew everything and he, sort of, wouldn’t listen. He just talked over us ... And I was, like, ‘‘we're here to tell you what the problem is; you don’t know us’’*.' (Travis)

Participants experienced increasing difficulty being heard as they became more unwell, and emphasised the importance of GPs facilitating, rather than avoiding, discussion about mental health concerns:

*'Don’t shy away from the illness of the person they’re trying to tell you ... You know, they don’t want to talk about it, but be ready to talk about it ... yeah. Not like, you know, not talk about it ... And be open about it. Because that will make the patients talk more. Talk more about how they’re feeling ... That’s why we come to the GP*.' (Patricia)

Several participants emphasised the importance of being asked the right questions to draw out important information which needed to be heard:

*'... you know, a few probing questions and bam, there you go it’s out and, you know, potentially you could save a life by doing that*.' (Carolyn)

Participants suspected that negative attitudes to mental illness underpinned practitioner reluctance to hear their mental health concerns, even in otherwise trusted GPs:

*'Even with my trusted GP, um, I trust him, but I don't think he’s comfortable with the whole mental health thing. He doesn't ask how I'm going, like, mentally. It’s mainly focused on physical health and he’s very good like that, but sometimes — it'd help if he felt more comfortable talking about mental health. I think — I don't know, I suspect that some people see it as some kind of weakness, that mental health is some kind of failing of the individual. So, I get that, sort of, weird kind of vibe from him. So, yeah. But I'm probably completely wrong* [laughs]. (Robert)

On the other hand, participants greatly appreciated GPs who listened respectfully and involved patients in unhurried and informed decisions:

*'I have so much time for my GP. He’s great ... he has always given me time to just talk with him about issues, um, and he’s been very non-judgmental during those times. And he offers good advice and he’s willing to accept my decision ... as a mental health patient, I think that is something that we crave ... I don’t think we like being left in the dark or being forced to do something that we don’t agree with. So, that’s why communication and involvement in decision making is so important for us*.' (Lee)

Being heard was contrasted with invalidating and dismissive encounters, which were also described:

*'I found sometimes that it is an attitude towards mental health where they think you're exacerbating it yourself, you're not perhaps making it up, but you’re attention seeking or you're blowing things out of proportion, that sort of thing ... It seems to be like, if you're suicidal, um, you've chosen to be suicidal ... people think that because it’s a way you feel, you must be able to function normally.'* (Emma)

### Being known

Many participants emphasised the importance of being known as a person by their GP, including their history of serious mental illness:

'[My GP] *now knows me more as a human being and it really is quite, um, you know, comforting to know that someone knows my story and I don’t have to keep repeating it*.' (Shy)

A practitioner who knew the extent of a patient’s illness might also detect early signs of a relapse, even before a patient was aware they were becoming unwell:

*'She obviously saw me at the lowest point so then she can see how you’re tracking as you’re, sort of, coming out of the deep end ... You do, kind of, need to have that relationship to be able to go, “Oh, hey, she walked in today just to ask for that but something doesn’t seem right, maybe I should probe that”, you know? I think that’s an important thing for a GP to have because you may the one that picks up on something first with the person not even knowing that it’s an issue*. (Caroline)

Participants became known over a number of consultations in which they experienced a sense of personal connection, or a ‘*friendly touch’*. They contrasted this with perfunctory care:

*'I suppose the only reason why I never went back to the other doctor was mainly because I didn’t feel like there was that sort of connection, I suppose. I just felt like it was just her job, and I was just her job, you know? And like it just makes me feel like a number, rather than a person*.' (Jen)

*'Just don’t, um, fall under the trap of just going through the motions of being a doctor ... just don’t fall in a rut and just, you know, go through the motions ... Yeah, have that bit of a friendly touch.'* (Ben)

Current or previous GPs were described by several participants as caring friends who knew them deeply:

*'He was a very good person I think ... I kind of considered him, um, uh, a friend you know. I could talk to him about things that were very uncomfortable ... He did, um, genuinely care about what was happening to me ... He knew what he was doing*.' (Patrick)

There were several mentions of practitioners who had themselves shared personal details with participants including, in one case, a lived experience of depression. Participants emphasised, however, that this was not the main purpose for the visit:

*'He would tell me about when he hiked the Kokoda trail and about a book he read ... that sort of thing, which was great, but I never came out with any answers ... I just really felt like he never took anything a step further, you know, he never followed up on anything, you know, no*.' (Ann)

Not all participants had experienced this depth of relationship, or sense of being known. Several commented that it was difficult to find *'good GPs*', and two participants were worried about finding a new practitioner when their current, long-standing GP retired. One participant, whose long-standing GP had retired, commented ironically that he had not sought to establish a similarly strong relationship with a new practitioner, hinting at the energy and effort required to be known in this way:

*'I think there’s various reasons I don't see* [GPs]*, but one of the big things is just simple anxiety. I just don't see anybody if I can help it ... it has to be very real and immediate reason for me to actually overcome that ... it’s difficult for me to, um, want to get to know somebody too well, ah, I think. I think that’s a bit of a barrier for me. So, in — in a way I'd sort of prefer just the hit and run* [laughs] *so to speak, with doctors*.' (Patrick)

### Being safe

Living with severe and persistent mental illness, and securing appropriate help, was a struggle for many participants:

*'I've struggled to get the help that I need and it sucks, because I know that I'm probably going to need help for the rest of my life to be able to deal with this. Um, and, not having faith, it’s like, why am I taking these pills? You know, I'm pumping all of these chemicals into my body, because somebody that I don't really trust has told me to. So, for me, it is a bit of a struggle*.' (Jenna)

Several participants tended to isolate and *'shut down*' as they became unwell, becoming increasingly reluctant to book and keep appointments. Participants wanted to feel safe, and one participant spoke of general practice as a safe haven in times of crisis:

*'You want to go to a GP where you can lie down and be treated. Not — not sit down wondering whether they’re going to give you a script or not ... I want a good, good GP that would say, ‘‘Well, at least you’re here. We don’t need to call the ambulance anymore. You’re safe with us’’ ... and when you, as soon as you enter the door it’s like, ‘‘Wow’’, you know, ‘‘I’m in a nice office’’. I can just sit here ... break away from the — the surroundings just for an hour. Just for an hour, you know*.’ (Hal)

In contrast to feeling safe, several interviews conveyed a constant fear of relapse:

*'I’m afraid of things falling through the gap. If things start to go sideways, you know. I have been diagnosed with bipolar II, and the last 2 years I have probably made four or more suicide attempts. I’ve been hospitalised in the mental health unit at least half a dozen times ... when things start going wrong with me, with my mental health, they’re extreme. So, it’s from zero to a 100 in a really short period of time. Um, now, I’ve been doing really well for over 6 months, I can’t foresee that happening, but it’s still in the back of my mind, like, if things do start going downhill again, am I going to be honest and will my GP see the signs? ... I’m just kind of scared that she won’t pick up the signs, because I hide it so well*.' (Ann)

Other participants were also uncertain whether they could trust their GPs in matters of mental health. Several emphasised the importance of practitioners reaching out for assistance when needed, and keeping up to date. Several were uncertain if GPs received mental health training:

*'I don't know if they're trained or not in mental health, but if they could communicate to the patient, um, schizophrenic or not, you could say, you know, ‘‘We've been trained in mental health. We can give you your medicines, you can come to us if you have an emergency’’ ... And also tried to get a bit up-to-date with drugs and stuff*.' (Robert)

Despite several comments on the GP role in the coordination and integration of their physical health care, there was little mention of such a role in their mental illness, except to facilitate access to medication or regular pathology tests. The consultation activities and tasks described by participants were related to physical health much more often than mental health. Several participants were more confident about their GP’s ability to keep them safe in matters of physical health, and some avoided involving them in mental health concerns:

*'She’s been our family GP, not just me but other members of my family for as long as I can remember. She’s just really helpful and she’s always um, straightforward in her approach ... she’s pretty much on top of everything, like she’s always sending me reminders for pap smears and mammograms and all that sort of stuff ... I should involve her more in my mental health but I haven’t*.' (Suzie)

Several participants acknowledged general practice workloads and time constraints, commenting that GPs could not spend enough time with patients to understand their mental health concerns (although others preferred relatively short appointments, commenting that 10–15 minutes was *'like, way long enough for me*' [Shy]). Several participants acknowledged the generosity of practitioners who did not charge them an out-of-pocket fee for consultations, but others believed that *'good GPs'* who could help with mental health issues were prohibitively expensive:

*'I know there are good GPs out there. It’s just not in the areas that are easy to get to. And trying to find a new GP is really hard ... the more richer and affluent suburbs have the good GPs ... you get what you pay for. You can either walk in and ask for a script and that’s it, over and done with and you get that for free, or you can walk in and ask for actual help and you've got to pay for it*.' (Jenna)

Participants were generally careful to convey reasonable expectations of GP care for people with serious mental illness:

*'If you’re really unwell, there’s only so much they can do ... like you can’t be God and fix me.'* (Sheila)

On the other hand, participants expected GPs to be competent at generating the various referrals, certificates, and other documents they required, and perhaps demanded on occasion. Two participants, for example, angrily recounted experiences of practitioners who declined to prescribe the medication they had requested, describing this ‘refusal’ as leading directly to a relapse:

*'He refused to give me any Valium to the point where it then led to another attack ... I just don't trust* [GPs] *... I'm very clear at what I want. And so for a GP to not give me what I want, there’s no excuse*.' (Jenna)

## Discussion

### Summary

The findings of these interviews show that many patients greatly value their personal connection with a regular GP. These patients emphasise the importance of being heard, being known, and being safe, and of a kind, respectful, and non-judgmental approach. Some patients have experienced perfunctory, transactional, or even dismissive approaches to their mental illness. Participants wanted their GPs to detect any relapse of their mental illness early, but an important and striking finding was the uncertainty many felt about trusting their knowledge in matters of mental health. Patients want GPs to ask about their mental health, and may suspect negative attitudes to mental illness in GPs who actively engage predominantly with their physical health.

While reflecting on these data, and their own responses to it, the authors noticed their sadness about the evident impacts of severe and persistent mental illness on participants, including the fear of sudden catastrophic symptom escalation and difficulties meeting typical expectations of normal functioning. The GP authors were also disappointed that ‘good’ general practice care sometimes seemed out of reach, and that experiences of perfunctory care seemed to be common. Indeed, patient expectations of care struck several of the authors as relatively low. It was noted that several participants were self-deprecating, acknowledging their difficulties with communicating and interpreting information, especially when more unwell. Many participants displayed considerable insight into the challenges facing practitioners caring for patients with severe and persistent mental illness. This may have been a tactic to avoid appearing critical, given participant vulnerability (including to involuntary treatment orders in several cases). The authors initially found it challenging to set aside their own responses so as to fully attend to participant voices, and found it useful to share and discuss these responses in some depth early in the analysis.

### Strengths and limitations

This project has brought the voices and perspectives of a diverse group of people with severe and persistent mental illness to the contested space of the role of the GP in their mental health care. GP perspectives were not sought, limiting any conclusions about the attitudes and beliefs of either ‘good GPs’ or those who were viewed less favourably. No attempt was made to measure patient outcomes, conduct repeat interviews, or check back with participants about the authors’ interpretations. No consultations were observed. This was a convenience sample, and patients with positive attitudes to general practice, or frequent attendance for GP care, may have been more likely to participate. Patients who appeared agitated or otherwise acutely unwell did not participate. Participants may have constructed their accounts to avoid appearing critical of general practice or clinic care. Participants were recruited from community mental health clinics. Although this was an effective strategy, findings may not transfer to patients who do not have access to this support and monitoring. Of note is that attendance at these clinics, especially by patients with psychosis, is relatively common in Australia (approximately 80% of patient with psychotic illness were estimated to attend these clinics in 2010).^20^ Another limitation of the research was that a conventional distinction between physical and mental health was assumed, and there was little exploration of participant understandings of ‘mental’ health and illness. Future research is indicated to explore these understandings further, and include the views of carers, GPs, and other practitioners involved with patients.

### Comparison with existing literature

The three themes of being heard, known, and safe have much in common with previously identified characteristics of deep or healing doctor–patient relationships. These characteristics include knowledge, loyalty, and regard,^21^ non-judgmental valuing and the careful management of power,^7^ and a sense of safety for patients with complex trauma.^6^ Trust has also been identified as key in this previous literature, highlighting the importance of the current finding that many participants did not trust their GP’s training and expertise (and in some cases, even their interest) in mental illness. Other literature has drawn attention to the alignment of general practice’s person-centredness and shared decision-making with a recovery paradigm, but questioned the extent to which the ‘*hopeful stance*’ and strengths-based approaches of this paradigm are seen in practice.^8^ The current project suggests that many GPs should consider increasing their emphasis on hope, strengths, and recovery for these patients.

Previous literature has drawn attention to the importance of screening, monitoring, and intervening in patients’ physical health issues.^9,22–25^ The current study, in contrast, highlights the potential of many GPs to play a more central role in mental health care and healing.

### Implications for research and practice

Some GPs play central roles in the whole-person care of patients with severe and persistent mental illness, and their generalist and relational skills are greatly valued by patients. Barriers for other GPs to engage more actively and effectively with these patients’ mental health need to be further explored and addressed: these barriers may include time, attitudes, confidence, expertise, and/or uncertainty about their role vis-à-vis other mental health practitioners. Findings suggest unrealised opportunities for long-term GPs to partner with patients in journeys of mental health healing and growth, rather than being content with more perfunctory, palliative, or ‘hands off’ approaches. Advocacy for the skillset and role of the GP within the mental health team, and further education and training of GPs by generalist champions with a special commitment to the care of these patients, may enable more GPs to engage holistically and effectively with primary care patients with severe and persistent mental illness.
